# Detecting a Novel Walking-Based Performance Fatigability Marker With Accelerometry in Older Adults

**DOI:** 10.1093/geroni/igab046.1301

**Published:** 2021-12-17

**Authors:** Yujia (Susanna) Qiao, Jaroslaw Harezlak, Robert Boudreau, Jacek Urbanek, Kyle Moored, Jennifer Schrack, Eleanor Simonsick, Nancy W Glynn

**Affiliations:** 1 University of Pittsburgh, University of Pittsburgh, Pennsylvania, United States; 2 Indiana University, Bloomington, Indiana, United States; 3 University of Pittsburgh, Pittsburgh, Pennsylvania, United States; 4 Johns Hopkins School of Medicine, Baltimore, Maryland, United States; 5 Johns Hopkins Bloomberg School of Public Health, Baltimore, Maryland, United States; 6 National Instute on Aging/NIH, Baltimore, Maryland, United States; 7 University of Pittsburgh Graduate School of Public Health, Pittsburgh, Pennsylvania, United States

## Abstract

Walking-based performance fatigability measures (e.g., lap-time difference) may not adequately capture performance deterioration as self-pacing is a common compensatory strategy in those with low activity tolerance. To overcome this limitation, we developed a new approach with accelerometry (ActiGraph GT3X+, sampling=80 Hz, non-dominant wrist) during fast-paced 400m-walk (N=57, age=78.7±5.7 years, women=53%). Cadence (steps/second) was estimated using raw accelerometer data (R “ADEPT”). Penalized regression splines (R “mgcv”) were used to estimate the individual-level smoothed cadence trajectories. “Time-to-slow-down” was defined as first time-point where the full confidence interval of change in cadence<0. Five participants were censored at stopping time (not slowdown or complete walk). Median “time-to-slow-down” was 1.86 minutes (IQR=0.98-2.73, range=0.57-6.25). Participants with longer “time-to-slow-down” had slower starting cadence, longer 400m-walk time, and greater perceived fatigability (Pittsburgh Fatigability Scale), p’s<0.05 (linear regression). Our preliminary findings revealed that detecting accelerometry-based performance fatigability/deterioration in older adults is feasible and needs to account for initial pace.

